# Association of heavy metals exposure with lower blood pressure in the population aged 8–17 years: a cross-sectional study based on NHANES

**DOI:** 10.3389/fpubh.2024.1411123

**Published:** 2024-07-05

**Authors:** Yongzhou Liang, Minjie Zhang, Wenhao Jin, Liqing Zhao, Yurong Wu

**Affiliations:** Department of Pediatric Cardiology, Xinhua Hospital, School of Medicine, Shanghai Jiao Tong University, Shanghai, China

**Keywords:** heavy metal, blood pressure, joint effect, children, NHANES

## Abstract

**Background:**

The existing evidence regarding the joint effect of heavy metals on blood pressure (BP) in children and adolescents is insufficient. Furthermore, the impact of factors such as body weight, fish consumption, and age on their association remains unclear.

**Methods:**

The study utilized original data from the National Health and Nutrition Examination Survey, encompassing 2,224 children and adolescents with complete information on 12 urinary metals (barium, cadmium, cobalt, cesium, molybdenum, lead, antimony, thallium, tungsten, uranium, mercury and arsenic), BP, and core covariates. Various statistical methods, including weighted multiple logistic regression, linear regression, and Weighted Quantile Sum regression (WQS), were employed to evaluate the impact of mixed metal exposure on BP. Sensitivity analysis was conducted to confirm the primary analytical findings.

**Results:**

The findings revealed that children and adolescents with low-level exposure to lead (0.40 μg/L, 95%CI: 0.37, 0.42), mercury (0.38 μg/L, 95%CI: 0.35, 0.42) and molybdenum (73.66 μg/L, 95%CI: 70.65, 76.66) exhibited reduced systolic blood pressure (SBP) and diastolic blood pressure (DBP). Conversely, barium (2.39 μg/L, 95%CI: 2.25, 2.54) showed a positive association with increased SBP. A 25th percentile increase in the WQS index is significantly associated with a decrease in SBP of 0.67 mmHg (95%CI, −1.24, −0.10) and a decrease in DBP of 0.59 mmHg (95% CI, −1.06, −0.12), which remains statistically significant even after adjusting for weight. Furthermore, among individuals who consume fish, heavy metals have a more significant influence on SBP. A 25 percentile increase in the WQS index is significantly associated with a decrease of 3.30 mmHg (95% CI, −4.73, −1.87) in SBP, primarily attributed to mercury (27.61%), cadmium (27.49%), cesium (17.98%), thallium (8.49%). The study also identified a declining trend in SBP among children aged 10–17, whereas children aged 11–18 exhibited lower levels of systolic and diastolic blood pressure, along with a reduced risk of hypertension.

**Conclusion:**

Some heavy metals demonstrate an inverse association with the BP of children and adolescents, particularly notable in groups with fish consumption and older children and adolescents. Future studies are warranted to validate these findings and delve deeper into the interplay of heavy metals.

## 1 Introduction

Hypertension is a well-known risk factor for cardiovascular diseases, with cardiovascular diseases remaining a leading cause of mortality in the United States (1). Over the last two decades, there has been a concerning upward trend in the prevalence of hypertension among children, with a relative growth rate ranging from 75 to 79% between 2000 and 2015 (2). Prolonged elevation of blood pressure (BP) in childhood may result in adult hypertension and increase the risk of cardiovascular diseases in adulthood (3, 4). Successfully reversing childhood hypertension before reaching adulthood could substantially decrease the susceptibility to cardiovascular diseases in the future (5). Hence, the prevention and management of childhood hypertension hold paramount significance in public health strategies.

The onset and progression of hypertension are believed to stem from intricate interactions among environmental factors, pathophysiology, and genetic susceptibility (6). In recent years, there is now a growing interest in the association between metals exposure and hypertension risk (7, 8). Studies have shown that exposure to arsenic (As), cadmium (Cd), lead (Pb), mercury (Hg), and barium (Ba) can increase the risk of hypertension, whereas molybdenum (Mo) is associated with reduced BP (9–12). However, epidemiological data on cobalt (Co), cesium (Cs), thallium (Tl), tungsten (W), and uranium (U) remains sparse, particularly in children and adolescents. Concurrently, research indicates a correlation between Ba exposure and elevated BP among children and adolescents (12). Although several studies have undeniably examined the impact of heavy metal exposure on BP in children, indicating that elevated concentrations of urinary antimony (Sb) might escalate BP, while Ba might exert the opposite effect (13, 14). The findings from epidemiological research regarding the association between environmental heavy metal exposure and the risk of childhood hypertension are inconsistent. Additionally, due to the enactment of diverse public health strategies, the levels of toxic metal exposure in the United States have decreased (15, 16). Given the lack of a clear threshold for the negative consequences of heavy metal exposure on health (17), it remains uncertain whether low-level exposure to multiple heavy metals is detrimental to BP among children and adolescents. Thus, further exploration of the association between heavy metal exposure and BP holds significant clinical and public health implications.

In research, it is necessary to consider the impact of dietary factors on the intake of heavy metals, particularly as certain fish species may accumulate high levels of heavy metals (18). Hence, the consumption of fish could potentially influence the association between heavy metals and BP in children (19). Moreover, as children age and undergo changes in weight, alterations occur in their physiological status and metabolic processes, resulting in variations in heavy metal exposure levels across different age and weight categories (14, 20).

Therefore, this study investigated the potential association of 12 urinary metal concentrations with the levels of BP as well as the risk of hypertension in 8-17-year-old individuals participating in the National Health and Nutritional Examination Surveys (NHANES). Various statistical models were used to study the effects of multiple metals on BP, conducting subgroup analyses to further explore the correlations between different groups.

## 2 Methods

### 2.1 Study population

This cross-sectional study focuses on children and adolescents aged 8–17 years. The data was obtained from U.S. NHANES cycles spanning from 2007 to 2016 (five consecutive NHANES cycles). The NHANES conducts a cross-sectional survey using a complex multistage probability design to collect data from the noninstitutionalized U.S. population. This involves conducting household interviews and physical examinations. Prior to participation, all individuals provided written informed consent, and the study protocol received approval from the NCHS Research Ethics Review Board. Out of the total of 9,386 participants, 7,162 had incomplete data, resulting in a final unweighted sample size of 2,224 individuals. The process of data acquisition is depicted in Supplementary Figure S1.

### 2.2 Blood pressure (BP)

In our study, participants who underwent BP measurements at the Mobile Examination Centers (MECs) were instructed to sit in a seated position with their feet flat on the floor and rest for a duration of 5 min. Trained researchers then performed three consecutive BP measurements using a mercury manometer and an appropriately sized cuff, which were subsequently averaged to obtain the final result (21).

The diagnostic criteria for hypertension were based on the 2017 clinical practice guidelines and previous studies (17, 22). The specific criteria for diagnosis include the following: (1) For children aged 8–12 years, hypertension is diagnosed when their systolic and/or diastolic blood pressure exceeds the 95th percentile compared to children of the same age, gender, and height. Alternatively, a diagnosis of hypertension can also be made if their SBP exceeds 130 mmHg and/or their DBP exceeds 80 mmHg. Conversely, for adolescents aged 13–17 years, hypertension is diagnosed if their SBP exceeds 130 mmHg and/or their DBP exceeds 80 mmHg (22); or (2) Irrespective of the BP level, patients aged ≥16 or the parents/guardians of patients aged <16 report the patient’s diagnosis of hypertension or the use of antihypertensive drugs (17, 23).

### 2.3 Urinary heavy metal collection and exposure assessment

In the NHANES, most metals were detected in urine samples rather than blood samples. The non-invasive, sensitive, and prompt detection capabilities of urine have increasingly positioned it as an alternative method for metal detection over blood samples. Therefore, all metal detection data in this study was based on urine samples. Upon arriving at the MECs, study participants were instructed by the coordinator to provide urine samples. Subsequently, urine samples undergo processing and analysis using inductively coupled plasma-mass spectrometry (ICP-MS) to determine the concentrations of 12 elements, including Ba, Cd, Co, Cs, Mo, Pb, Sb, TI, W, U, Hg, and As.^1^ Comprehensive instructions for laboratory methods utilized to measure the urinary metal concentrations can be found on the NHANES website.^2^ In our statistical analysis, the concentrations of urinary metals were used as a variable after being transformed with natural logarithm (Ln). Approximately 70% of the cleared As from the blood is excreted through urine. Thus, we opted to assess As exposure by measuring the total concentration of As in urine, rather than focusing on speciated As. Moreover, these data pertain to the overall healthy population, and restricting the analysis to speciated As may lead to an underestimation of long-term exposure levels. Nonetheless, this study conducted further analysis on speciated As, namely arsenobetaine (AsB), arsenic acid (As(V)), arsenocholine (AsC), arsenous acid (As(III)), monomethylarsinic acid (MMA), and dimethylarsinic acid (DMA). Exclusion of trimethylarsine oxide from the analysis was due to the unavailability of subject data for NHANES 2013–16 cycle (24).

### 2.4 Covariates

Based on previous literature, several covariates were extracted as potential confounding factors in this study (17, 25, 26). The selected covariates included age, sex, race/ethnicity (Mexican American, other Hispanic, non-Hispanic White, non-Hispanic Black, and other race), family poverty income ratio (PIR, categorical variables: <1.3, 1.3–3.5, >3.5 denote low, middle and high income, respectively), serum creatinine (an indicator of renal function), urinary creatinine (Detection method: Enzymatic Roche Cobas 6,000 Analyzer) and serum cotinine levels (25) (considered to reflect exposure to environmental cigarette smoke). Physical activity was fell into three groups (never, moderate or vigorous, and no record) according to self-reported activity intensity. Consistent with a previous study, the population was divided into three age groups: 8–10 years old, 11–13 years old, and 14–18 years old, representing primary school, junior high school, and senior high school, respectively (27). Additionally, the consumption of fish in the past 30 days (Yes or No) was included as a covariate in the model to account for the impact of dietary factors on urinary heavy metal concentrations. Body mass index (BMI) was calculated using the formula weight (in kilograms) divided by the square of height (in meters). Underweight was an age-and gender-specific BMI below the 5th percentile on the 2000 Centers for Disease Control and Prevention (CDC) age-and gender-specific growth charts, normal weight was a BMI below the 85th percentile but at or above the 5th percentile, overweight was a BMI falling between the 85th and 95th percentiles, and obesity was a BMI at or above the 95th percentile (28).

Additionally, all participants in the NHANES are eligible for two separate 24-h dietary recall interviews. Nonetheless, fewer participants had completed two 24-h dietary recalls. Consequently, the research evaluated the daily intake of total energy, calcium, sodium, and potassium using data from the first recall (23), which were then incorporated as covariates in our analysis.

### 2.5 Statistical analysis

We utilized the NHANES weighting guidelines to weigh the analysis results. Our study incorporated sample weights, sampling units, and strata provided by NHANES. We combined two groups of 5 years to conform two periods for the calculation of a new multiyear sample, ensuring that the results represent the nationwide non-institutionalized civilian population aged 8–17 years. In descriptive analysis, means ± standard deviation (SD) and counts (percentage) are applied to, respectively, describe quantitative and qualitative data. Spearman’s rank correlation analysis was performed to examine the correlations of urinary toxicant concentrations. Subsequently, due to the presence of values below the detection limit for certain individuals, urinary toxicant concentrations were categorized into four quartiles (Q1, Q2, Q3, Q4) as categorical variables, with the quartile containing the lowest metal concentrations serving as the reference group. Survey-weighted logistic regression and survey-weighted multiple linear regression models were employed to calculate odds ratios (ORs), β, and 95% confidence intervals (CIs). The false-discovery rate (FDR) correction was applied to adjusted for errors resulting from multiple testing in regression models.

The WQS approach has been widely utilized to investigate the cumulative effect of environmental mixtures on health outcomes and to assess the contribution of individual metals (29, 30). The urinary metals composing a weighted index were divided into quartiles and then applied in the estimation of empirically deduced weights and a final WQS index by bootstrap sampling (31, 32). This WQS index denotes the cumulative effect of all urinary toxicants on BP. The weights sum to 1 and range from 0 to 1, and they can be applied to identify important urinary metals (the average weight surpasses the threshold of 1 divided by the total number of independent variables) in the mixture (33). The WQS index was constructed from the quartiles of urinary metals, with 40% of participants in this study divided into the test set and 60% into the validation set. Initially, we examined whether the relationship between heavy metal concentration and BP is influenced by body weight by adjusting the BMI category in the WQS model. Subsequently, we divided the samples into two groups depending on fish consumption in the past 30 days to investigate the impact of fish consumption on the association between heavy metals and BP. Lastly, to investigate the impact of heavy metals on BP in children and adolescents across different age groups, we categorized the age of pediatric patients into seven time intervals based on previous research (34). The first group comprised individuals aged 8–14, the second group included those aged 9–15, and so on, in order to analyze the trend of the effect of heavy metal concentration on BP with changing age.

In our sensitivity analysis, we initially considered the potential non-linear and non-additive relationships among urine metals. Bayesian kernel machine regression (BKMR) was employed to assess the combined effects of all metals and the dose–response relationship between individual metals and BP when fixing other metal concentrations. Secondly, participants were excluded if their urine samples were categorized as either diluted (urine creatinine <30 mg/dL, *n* = 139) or concentrated (urine creatinine concentration > 300 mg/dL, *n* = 63), according to previous study (26, 35). Subsequently, WQS analysis was conducted.

All statistical analyses were performed with R statistical software (V.4.4.0),^3^ and a two-sided *p* value <0.05 was considered statistically significant. The R packages gWQS and nhanesR were applied to construct the WQS model, weighted logistic regression and multiple linear regression, respectively.

## 3 Results

### 3.1 Baseline characteristics of the participants

Supplementary Figure S1 illustrates the process of acquiring the study population. Table 1 and Supplementary Table S1 present the weighted general characteristics and urinary metal concentrations of 2,224 children and adolescents aged 8–17 years from NHANES 2007–2008 to 2015–2016. The weighted average age of the participants was 12.81 ± 0.08 years, with male and female proportions of 50.63 and 49.37%, respectively. The overall prevalence of hypertension was 10.48% (*n* = 233). Most of the participants were Mexican American and had attained higher education levels. Among hypertension patients, there was a higher proportion of individuals who were overweight or obese, had increased daily energy and sodium intake, and a lower proportion of those with unavailable serum creatinine values. Additionally, both systolic and diastolic blood pressures were elevated, while no significant differences were observed in other general characteristics. In comparison to children and adolescents without hypertension, hypertensive patients exhibited lower concentrations of Co (0.56 μg/L, 95%CI, 0.49, 0.63), Cs (4.37 μg/L, 95%CI, 3.97, 4.78), TI (0.18 μg/L, 95%CI, 0.16, 0.20), and As (8.74 μg/L, 95%CI,6.62, 10.86) in their urine samples, whereas there were no significant differences in the concentrations of Ba (2.38 μg/L, 95%CI, 1.82, 2.94), Cd (0.08 μg/L, 95%CI, 0.07, 0.10), Mo (75.31 μg/L, 95%CI, 65.28, 85.33), Pb (0.38 μg/L, 95%CI, 0.32, 0.43), Sb (0.08 μg/L, 95%CI, 0.07, 0.09), W (0.19 μg/L, 95%CI, 0.15, 0.22), U (0.02 μg/L, 95%CI, 0.00, 0.03), and Hg (0.38 μg/L, 95%CI, 0.29, 0.46). The proportions of urinary Cd and Hg concentrations exceeding the limits of detection (LOD) were the lowest, at only 65.47 and 76.35%, respectively, whereas the proportions of other urinary metals exceeding the LOD were all above 80% (Supplementary Table S1).

**Table 1:** Distribution of general characteristics of in children and adolescents in NHANES 2007–2016.

Characteristic	Overall	No-hypertension	Hypertension	*p*-value
	(*n* = 2,224)	(*n* = 1991)	(*n* = 233)	
Mean ± SD^a^				
Age, years	12.81 ± 0.08	12.85 ± 0.09	12.41 ± 0.24	0.100
Cotinine, ng/ml	5.89 ± 1.07	5.58 ± 1.08	8.78 ± 3.96	0.430
SBP, mmHg	105.69 ± 0.33	104.27 ± 0.31	118.80 ± 0.81	**< 0.001**
DBP, mmHg	57.69 ± 0.43	56.96 ± 0.40	64.45 ± 1.31	**< 0.001**
Urinary creatinine, μg/L	121.60 ± 2.06	122.48 ± 2.13	113.52 ± 6.68	0.200
Total energy, kcal/day	2092.83 ± 25.54	1962.84 ± 47.84	2106.90 ± 28.23	**0.020**
Calcium intake, mg	1063.62 ± 20.13	1010.67 ± 43.19	1069.35 ± 22.61	0.260
Sodium intake, mg	3361.77 ± 52.46	3145.20 ± 98.43	3385.21 ± 55.96	**0.030**
Potassium intake, mg	2234.94 ± 27.50	2169.04 ± 65.84	2242.07 ± 29.09	0.300
*N* (%)^b^				
Sex				0.970
Female	1,098(49.37)	989(49.91)	109(49.70)	
Male	1,126(50.63)	1,002(50.09)	124(50.30)	
Race/ethnicity				0.060
Mexican American	638(55.78)	579(56.40)	59(50.07)	
Other Hispanic	536(14.82)	469(14.38)	67(18.81)	
Non-Hispanic White	521(13.63)	455(13.12)	66(18.28)	
Non-Hispanic Black	281(8.94)	259(9.17)	22(6.86)	
Other race	248(6.83)	229(6.92)	19(5.99)	
Obesity				**0.002**
Underweight	60(2.7)	57(2.52)	3(0.74)	
Normal weight	1,271(57.15)	1,161(57.73)	110(47.04)	
Overweight	380(17.09)	338(18.22)	42(16.52)	
Obesity	513(23.07)	435(21.53)	78(35.70)	
PIR				0.920
Low income	950(42.72)	841(30.02)	109(35.30)	
Middle income	807(36.29)	715(37.91)	92(40.72)	
High income	467(21)	435(32.08)	32(23.98)	
Education				0.080
Primary school	686(30.85)	602(24.82)	84(33.34)	
Junior high school	644(28.96)	581(27.75)	63(27.34)	
Senior high school	894(40.2)	808(47.42)	86(39.32)	
Fish consumption				0.760
No	1,271(57.15)	1,129(59.76)	142(61.06)	
Yes	953(42.85)	862(40.24)	91(38.94)	
Activity				0.580
Never	109(4.74)	97(4.70)	12(5.14)	
Moderate or vigorous	217(11.97)	197(12.23)	20(9.53)	
No record	1898(83.30)	1,697(83.08)	201(85.33)	
Serum creatinine				**0.020**
No	952(42.81)	838(34.38)	114(44.76)	
Yes	1,272(57.19)	1,153(65.62)	119(55.24)	

Complex sampling weights were used for the results.

SBP, systolic blood pressure; DBP, diastolic blood pressure; PIR, family poverty income ratio; SD, standard deviation.

^a^Weighted mean ± weighted SD.

^b^Sample size (weighted percentage).

Bold values indicate statistical significance *p* < 0.05.

### 3.2 Correlation of the urinary metals

Supplementary Figure S2 displays weak to moderate correlations (−0.02 ≤ r ≤ 0.67) among all toxic metals, as calculated using Spearman’s rank correlation analysis. Cs and TI exhibit the strongest correlation (*r* = 0.67, *p* < 0.05), followed by Mo and W, while the correlation between Cd and W is the weakest. Consequently, it may be necessary to construct a multi-pollution model to detect the impact of toxic metals on BP.

### 3.3 Association of single metal exposure with blood pressure

Survey-weighted logistic regression and multiple linear regression models were utilized to investigate the association between Ln-transformed urinary metal concentrations and BP. These models were adjusted for selected potential confounding factors. The results presented in Supplementary Table S2 indicate a null significant correlation between urinary metal concentrations and hypertension (all *p* for trend >0.05). Supplementary Table S3 revealed a negative association between an increase in urinary concentrations of Pb (*p* for trend =0.036), Hg (*p* for trend =0.036) and SBP. Moreover, an increase in Ln-transformed Mo concentration was associated with a decreasing trend in DBP (*p* for trend <0.001). However, for the remaining urinary metal concentrations, no significant trend effects on BP were observed in the single-metal models.

### 3.4 Association of urinary metal co-exposure with blood pressure

**Figure 1:**
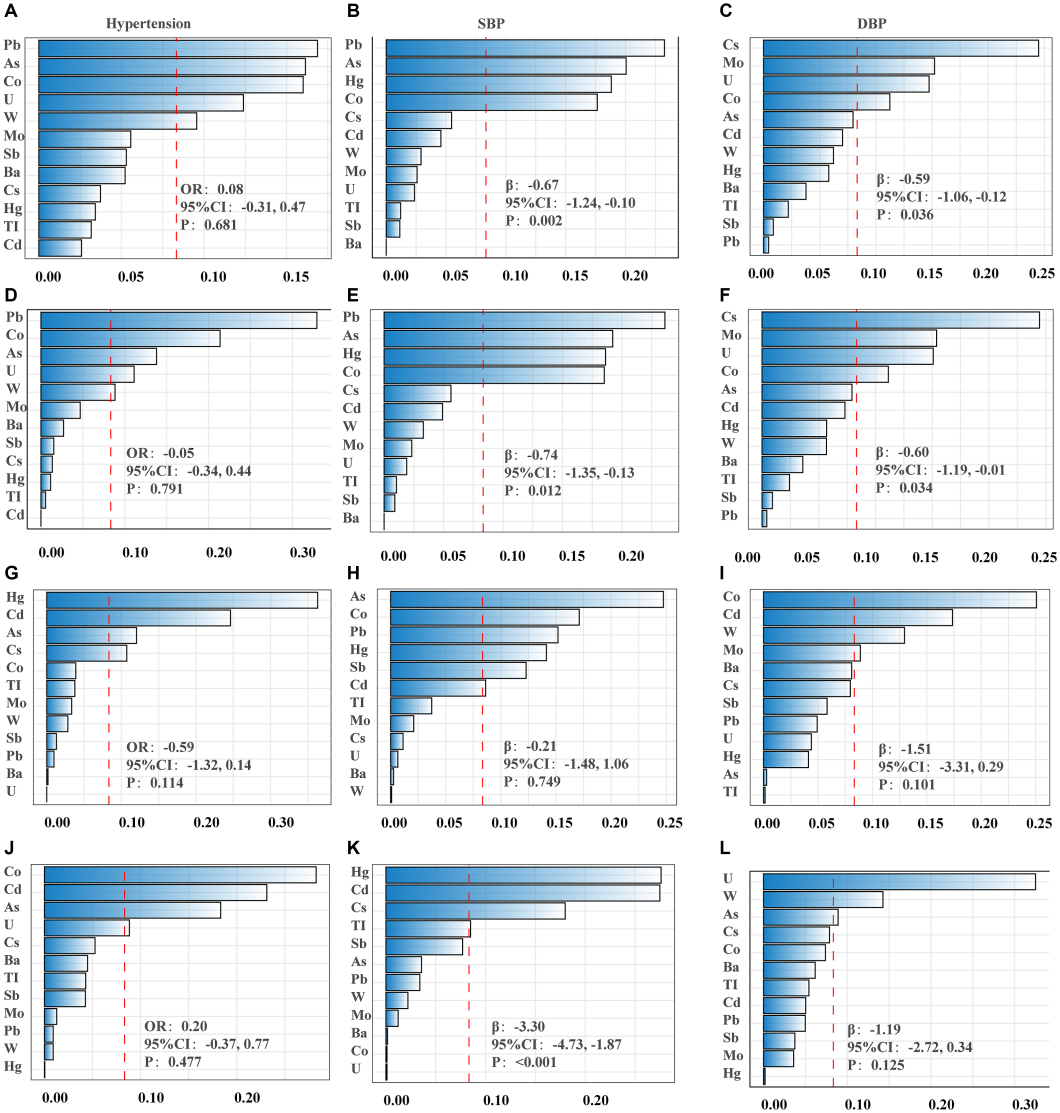
The weights of each metal in WQS model regression index for blood pressure. **(A–C)** Models were adjusted for sex, age, race/ethnicity, family poverty income ratio (PIR), obesity, education, serum cotinine, serum creatinine, urinary creatinine, fish consumption, total energy, calcium intake, sodium intake, potassium intake and activity. **(D–F)** Obesity was excluded from the models. **(G–I)** Non-fish consumers. **(J–L)** Fish consumers. **(A,D,G,J)** Presented hypertension; **(B,E,H,K)** presented systolic blood pressure (SBP); **(C,F,I,L)** presented diastolic blood pressure (DBP).

The WQS regression model was employed to examine the impact of mixed metals on BP. After adjusting for all selected confounding factors, null association was observed between low concentrations of urinary metal mixtures and the risk of hypertension (OR_index_: 0.08, 95%CI: −0.31, 0.47, *p* = 0.681) (Table 2; Figure 1A). Conversely, these mixtures were correlated with lower SBP (β_index_: −0.67, 95%CI: −1.24, −0.10, *p* = 0.002) and DBP (β_index_: −0.59, 95%CI: −1.06, −0.12, *p* = 0.036) (Table 2). Metal mixtures primarily affected SBP through Pb (23.62%), As (19.22%), Hg (18.62%), and Co (18.52%) (Figure 1B), while the effects on DBP were primarily attributed to Cs (24.48%), Mo (15.22%), U (14.74%), and Co (11.24%) (Table 2; Figure 1C). Furthermore, excluding the degree of obesity from the model yielded similar results to the obesity-corrected model (Figures 1D–F). Subgroup analyses were conducted to determine how fish consumption influenced the impact of heavy metals on BP. The analyses were based on participants’ fish consumption in the past 30 days. The results indicated that heavy metal exposure did not significantly affect BP values in the subgroup without fish consumption (Figures 1G–I). However, in the subgroup with fish consumption, heavy metals were associated with lower SBP (β_index_: −3.30, 95%CI: −4.73, −1.87, *p* ≤ 0.001) (Table 2; Figures 1J–L). The main contributors were Hg (27.61%), Cd (27.49%), Cs (17.98%), and TI (8.49%) (Table 2; Figure 1K).

**Table 2:** Association of WQS indices with blood pressure.

	WQS mixture result^a^	*p*-value	Component (weights)^b^
Hypertension			
Un-adjusted by obesity	−0.05(−0.34, 0.44)	0.791	NA
Adjusted by obesity	0.08(−0.31, 0.47)	0.681	NA
Not fish consumption	−0.59(−1.32, 0.14)	0.114	NA
Fish consumption	0.20(−0.37, 0.77)	0.477	NA
SBP			
Un-adjusted by obesity	−0.74(−1.35, −0.13)	**0.012**	Pb (23.26%), As (20.05%), Hg (18.82%), Co (17.64%)
Adjusted by obesity	−0.67(−1.24, −0.10)	**0.002**	Pb (23.62%), As (19.22%), Hg (18.62%), Co (18.52%)
Not fish consumption	−0.21(−1.48, 1.06)	0.749	NA
Fish consumption	−3.30(−4.73, −1.87)	**<0.001**	Hg (27.61%), Cd (27.49%), Cs (17.98%), TI (8.49%)
DBP			
Un-adjusted by obesity	−0.60(−1.19, −0.01)	**0.034**	Cs (24.45%), Mo (15.39%), U (15.09%), Co (11.13%)
Adjusted by obesity	−0.59(−1.06, −0.12)	**0.036**	Cs (24.48%), Mo (15.22%), U (14.74%), Co (11.24%)
Not fish consumption	−1.51(−3.31, 0.29)	0.101	NA
Fish consumption	−1.19(−2.72, 0.34)	0.125	NA

^a^The estimated parameters (OR_index_ or β_index_) for the metal mixture in each model, along with the corresponding 95% confidence interval (CI) and *p*-value, were reported. OR_index_ indicates the change in risk of hypertension corresponding to a 25th percentile increase in the WQS index, whereas β_index_ represents the change in blood pressure associated with a 25th percentile increase in the WQS index.

^b^The component (weights) represent the metals in the model that exhibit significant effects and indicate their corresponding percentages.

Bold values indicate statistical significance *p* < 0.05.

SBP, systolic blood pressure; DBP, diastolic blood pressure; NA, not applicable.

Following the consideration of potential confounding factors, the impact of urinary heavy metals on the BP of children and adolescents across various age groups was investigated using the WQS regression model. The findings are outlined as follows: within the 10–17 age group, a 25th percentile increase in the WQS index corresponded to a 1.48 mmHg (95% CI, −2.66, −0.30) reduction in SBP. Meanwhile, in the 11–18 age group, each 25th percentile rise in the WQS index led to reductions of 1.42 mmHg (95% CI, −2.44, −0.40) in SBP and 2.62 mmHg (95% CI, −4.00, −1.22) in DBP, contributing to a 0.42 times (95% CI, −0.81, −0.03) decrease in hypertension risk. Notably, no significant association between heavy metal exposure and BP was observed among the 8–15 and 9–16 age groups (Table 3).

**Table 3:** Trend analysis of the combined effect (WQS indices) of metal mixtures.

Ranges of age	WQS mixture result	*p*-value
	Hypertension, OR (95%CI)	
8 ~ 15	0.02 (−0.43, 0.47)	0.940
9 ~ 16	−0.02 (−0.51, 0.47)	0.591
10 ~ 17	−0.39 (−0.84, 0.06)	0.173
11 ~ 18	−0.42 (−0.81, −0.03)	**0.021**
	SBP, β (95%CI)	
8 ~ 15	−1.01 (−2.09, 0.07)	0.065
9 ~ 16	−1.18 (−2.40, 0.04)	0.061
10 ~ 17	−1.48 (−2.66, −0.30)	**0.013**
11 ~ 18	−1.42 (−2.44, −0.40)	**0.007**
	DBP, β (95%CI)	
8 ~ 15	−1.29 (−2.70, 0.12)	0.071
9 ~ 16	−1.35 (−2.90, 0.20)	0.089
10 ~ 17	−1.01 (−2.08, 0.07)	0.065
11 ~ 18	−2.62 (−4.00, −1.22)	**<0.001**

Association of WQS index with blood pressure in different age groups was reported, presented as OR_index_ or β_index_ (95% confidence interval, 95%CI).

Bold values indicate statistical significance *p* < 0.05.

SBP, systolic blood pressure; DBP, diastolic blood pressure.

**Figure 2:**
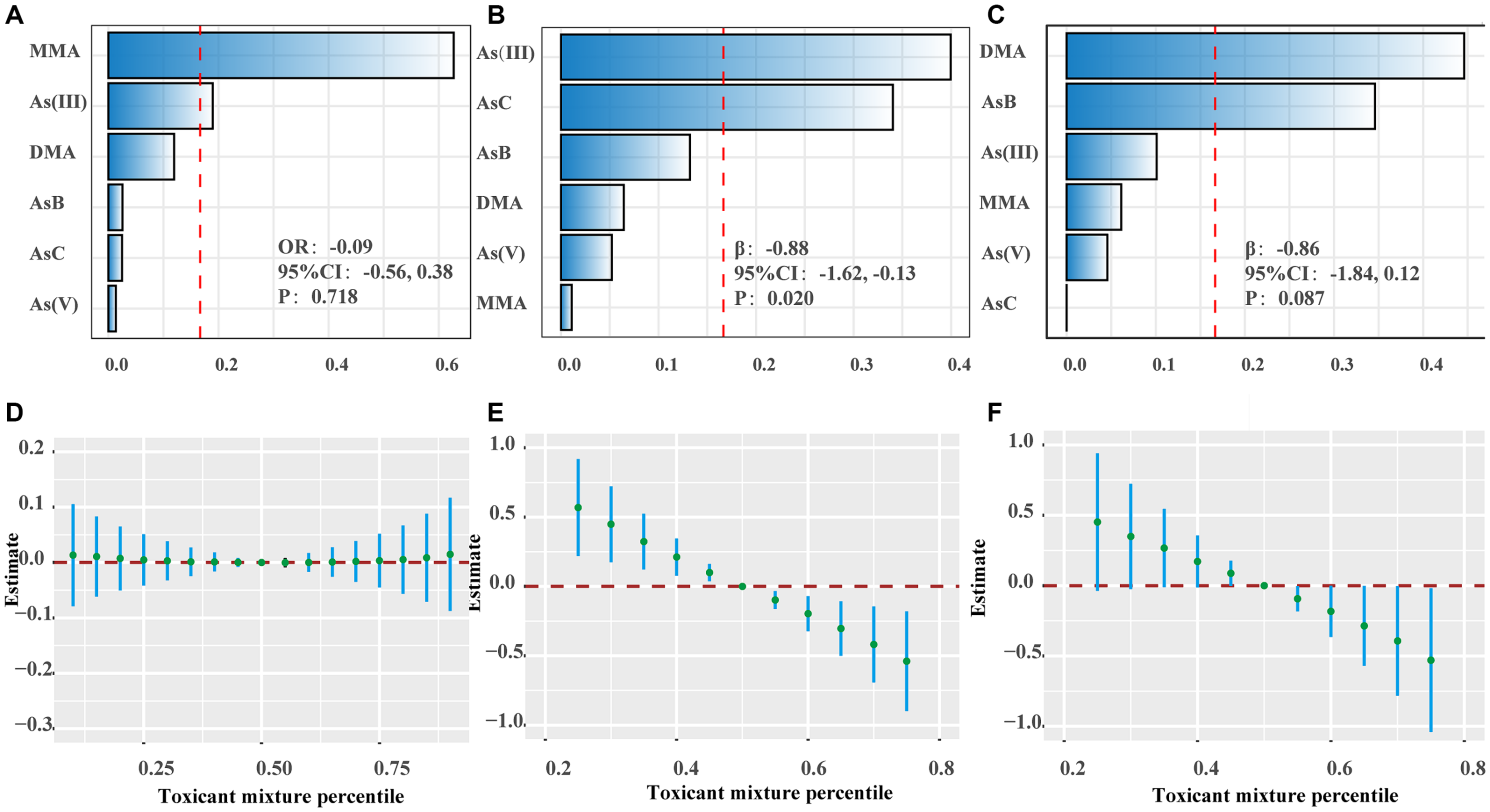
The effects of speciated As and metal mixtures on blood pressure. **(A–C)** The weights of speciated As in WQS model regression index for hypertension, systolic blood pressure (SBP) and diastolic blood pressure (DBP). **(D–F)** Joint effects of metal mixtures on hypertension, SBP and DBP using Bayesian kernel machine regression (BKMR). The results were adjusted for sex, age, race/ethnicity, family poverty income ratio (PIR), obesity, education, serum cotinine, serum creatinine, urinary creatinine, fish consumption, total energy, calcium intake, sodium intake, potassium intake and activity.

To examine the association between speciated As and BP, we adopted the WQS to model these categories, comprising AsB, As(V), AsC, As(III), MMA, and DMA (Supplementary Table S4; Figures 2A–C). A 25th percentile increase in the WQS index was associated with a decrease in SBP by 0.88 mmHg (95% CI, −1.62, −0.13), predominantly influenced by As (III) (40.00%) and AsC (34.04%) (Supplementary Table S5).

### 3.5 Sensitivity analysis

The results of the BKMR model reveal that when utilizing the median concentration (50th percentile) of all metals as the reference exposure level, a concentration of the mixture at or above the 55th percentile is associated with a downward trend in both systolic and diastolic blood pressure (Figures 2D–F). Furthermore, regardless of the percentile level (25th, 50th, or 75th) of other metals, Ba exhibits a significant association with elevated SBP (Supplementary Figure S3). The analysis yielded consistent results with the preliminary analysis, not just in the subgroups analyzed based on fish consumption, but also among participants with a urine creatinine concentration ranging between 30 mg/dL and 300 mg/dL (Supplementary Table S6; Supplementary Figures S4, S5).

## 4 Discussion

Several studies provide undeniable epidemiological evidence supporting the link between heavy metal exposure and BP in children and adolescents (12–14). This study is innovative in its investigation of the potential influence of weight and fish consumption on the relationship between heavy metal exposure and BP, and in exploring whether the effects of heavy metal exposure on BP differ across different age groups. The study employed both single metal and mixed metal chemical analysis models to assess the impact of urinary heavy metal concentrations on BP in children and adolescents. The findings are as follows: (1) Single-metal analysis indicated that Pb and Hg were associated with lower SBP. Likewise, Mo demonstrated a reduction in DBP. Moreover, the findings from the analysis of mixed metals further substantiated the impact of Pb, Hg, and Mo on BP reduction. Nevertheless, the BKMR model revealed that Ba was associated with an elevation in SBP; (2) Mixed metal exposure led to reduced systolic and diastolic pressures regardless of participants’ body weight; (3) Consumers of fish exhibit lower SBP, mainly attributed to exposure to Hg (27.61%), Cd (27.49%), Cs (17.98%), and TI (8.49%); (4)The effects of heavy metal exposure on BP varied across different age groups. A downward trend in SBP was noted in children aged 10–17, while children aged 11–18 exhibited lower SBP and DBP, along with a reduced risk of hypertension.

It is well known that lifestyle-risk factors can affect BP, but an increasing number of studies have implicated heavy metals as a latent risk factor for hypertension (7, 36). Heavy metals are naturally existing substances widely distributed in the environment and are widely used in industry, households, agriculture, medicine and other fields (37). Children and adolescents are inevitably exposed to these metals simultaneously in their daily activities, of which the most prominent are Pb, Cd, and Hg (22). Our study revealed an association between Pb (0.40 μg/L, 95%CI, 0.37, 0.42), Hg (0.38 μg/L, 95%CI, 0.29, 0.46), Mo (75.31 μg/L, 95%CI, 65.28, 85.33) and lower BP in both the single metal chemical model and the mixed metal chemical model, providing further evidence of children and adolescents’ susceptibility to Pb and Hg exposure in their daily activities. Additionally, the BKMR model exhibits an association between Ba (2.39 μg/L, 95%CI, 2.25, 2.54) and higher SBP.

Currently, there is inconsistent research evidence regarding the relationship between Pb，Hg and BP. A study in in Brazilian adults revealed that blood Pb levels (1.97 μg/dL, 95% CI, 1.90–2.04 μg/dL) were correlated with elevated DBP and an augmented hypertension risk (38). Conversely, a study focusing on children and adolescents showed no significant relationship between urinary Pb levels (0.31 μg/L, IQR, 0.18–0.57) and systolic or diastolic blood pressure (17). Another study with children and adolescents found no association between blood Hg (From 0.52 to 0.74 μg/L) and SBP. Nonetheless, a correlation was observed between total Hg and methylmercury, resulting in a DBP reduction (17). Conversely, data from adult epidemiological studies suggests that urinary Hg (0.433 μg/g, 95% CI, 0.400–0.469) was related to hypertension (9, 10). Our study diverges from these findings. Initially, we selected urine samples as the biomarker for assessment. Additionally, our study focuses on children and adolescents as the target population, who may exhibit lower exposure levels. Furthermore, this trend may stem from the limited focus in most studies solely on single metal analyses. In fact, the accumulation of Pb and Hg in the human body promotes arterial atherosclerosis through various mechanisms such as lipid peroxidation, vascular inflammation, endothelial dysfunction, and inhibition of nitric oxide (39, 40). Pb appears to activate the adrenergic system, potentially altering arterial tension, and it may also activate endothelin, resulting in vasoconstriction (40). Consequently, these pathophysiological consequences result in elevated SBP (39).

A study conducted on children and adolescents has demonstrated that a two-fold increase in urinary Ba concentration was associated with a rise of 0.41 mmHg in SBP and 1.04 mmHg in pulse pressure (12). Ba, by means of oxidative stress and inflammation, induces a reduction in the activity of nitric oxide synthase and the bioavailability of nitric oxide (NO), leading to endothelial dysfunction, heightened systemic vascular resistance, and SBP (41, 42). As an essential trace element, Mo is a cofactor of a variety of metabolic enzymes, including xanthine oxidase, sulfite oxidase and nitrate reductase (43). Studies have demonstrated that molybdate and metabolic enzymes containing Mo can improve vascular smooth muscle contraction, leading to a decrease in BP by reducing oxidative stress, enhancing nitric oxide (NO) synthesis, and promoting the release of vascular prostanoid (a vasodilator) (44–46). These findings align with our research results, supporting the conclusion that Ba is capable of inducing an elevation in BP, whereas low concentrations of Mo exhibit a hypotensive effect.

Recent studies have shown a positive association between urinary heavy metal exposure and obesity in children and adolescents (34). A study investigating the impact of heavy metal exposure on hypertension revealed that participants with a BMI ≥ 30 exhibited a positive association between heavy metal exposure and hypertension, whereas no association was found among those with a BMI < 30 (20). Furthermore, within the normal weight range for children and adolescents, the study found that a BMI ranging from the 25th to the 84th percentile was positively associated with higher BP and an increased risk of hypertension (47). These findings suggest that body weight acts as a significant confounding factor in the relationship between heavy metal exposure and BP. However, our study revealed that, even after adjusting for obesity, heavy metal exposure exhibited a significant association with lower BP, indicating its independence from body weight in children and adolescents. Further investigation is required to determine the specific mechanisms underlying this relationship.

Fish helps establish a cardioprotective dietary pattern, as advocated by the Mediterranean and Dietary Approaches to Stop Hypertension (DASH) diet (48, 49). Fish provides essential nutrients such as iodine, selenium, vitamin D, and ω-3 long-chain polyunsaturated fatty acids, but it also poses risks of Hg and organic As exposure (50). Reports have suggested that As can induce hypertension through oxidative stress, inflammation, and endothelial dysfunction (51). Our study revealed that the reduction in BP observed in individuals who consume fish is primarily attributed to Hg, suggesting that some fish types can increase Hg exposure. Although there is a correlation between As exposure and decreased SBP, this connection was not evident in fish consumers. To investigate the antihypertensive effects of As, we examined the correlation between speciated As and BP, in addition to total urinary As. Analysis of speciated As including AsB, As(V), AsC, As(III), MMA, and DMA revealed the significant roles of As(III) (40.00%) and AsC (34.04%) in WQS model. However, the detection rate of AsC was only 6.43%, which might not fully account for its impact on BP. Previous research has confirmed the association between As(III) and the risk of hypertension (52). Notably, the single-metal model indicated a relationship between Pb and Hg exposure and decreased SBP. The BKMR model demonstrated associations of Hg, and As with reduced SBP, and Ba with increased SBP. Furthermore, Pb, Hg and As contributed to lowered SBP in the WQS model, but these findings were inconsistent. Previous studies have highlighted that Ba, Pb, and Hg primarily cause hypertension through oxidative stress, inflammation, and endothelial dysfunction (9, 10). Surprisingly, our study revealed a connection between Pb, Hg, As, and decreased BP, contrary to previous studies. In mice, exposure to low levels of methylmercury notably raised plasma renin levels, leading to elevated BP, whereas co-exposure to Pb and Hg reversed this effect (10, 53). These results suggest antagonistic interactions between mixed metal components, aiding in the understanding of our findings. Overall, consuming specific types of fish can lead to heightened exposure to some heavy metals, which warrants further research.

This study confirms that low-level exposure to heavy metals is associated with lower SBP and DBP in older children and is also linked to a lower risk of hypertension. However, the study did not attempt to investigate the long-term effects of chronic heavy metal exposure on BP. More precisely, it only emphasizes whether there are differences in the effects of heavy metal exposure on BP among children and adolescents of different age groups. This limitation arises because most metals detected in urine reflect recent exposure. For instance, inorganic As is excreted within 4 days, Ba is mostly eliminated within 3–42 days after exposure, and the half-life of Hg is generally no longer than 3 months (50). Currently, research on the impact of heavy metal exposure on BP in different age groups of children and adolescents is relatively limited. A previous study focused on preschool children, showing no significant association between urinary Ba concentration and DBP among different age groups (ages 2–3, 4, 5, and 6 years) (14). Additionally, an animal experimental study has indicated that Pb exposure during infancy disrupts bone metabolism, with more noticeable effects on bone microstructure compared to childhood and adolescence (54). Similarly, these findings suggest the presence of an important critical period for the effects of heavy metal exposure on BP in children and adolescents, but the specific mechanisms need further elucidation.

This study examined the potential association between urinary metal concentrations and BP as well as hypertension risk in children and adolescents using diverse statistical models. Inevitably, this study has several limitations. Initially, following the methodology of prior studies where missing covariates were coded as categorical variables (55), we categorized the serum creatinine into two groups: available and unavailable. While this study incorporated urinary creatinine, it may not offer an optimal solution, necessitating further investigation in future research. Moreover, covariates associated with BP, such as dietary factors (excluding fish), were not completely controlled in this study. Additionally, as a cross-sectional study, it prevents drawing causal inferences that metal exposure causes BP changes. Further studies with a prospective design are necessary to validate these discoveries. Furthermore, given the complex composition of metal mixtures in the environment, it is essential to elucidate the synergistic and antagonistic mechanisms among heavy metals to regulate each metal at its optimal exposure level.

## 5 Conclusion

Our study revealed a negative correlation between low-level heavy metal exposure and BP in children and adolescents, particularly notable in groups with fish consumption and older children and adolescents. In future research, validating our findings through a prospective cohort study, elucidating the potential interactive mechanisms of heavy metals, and specifying the possible windows of susceptibility affecting childhood BP are crucial.

## Data availability statement

The original contributions presented in the study are included in the article/Supplementary material, further inquiries can be directed to the corresponding authors.

## Ethics statement

Ethical approval was not required for the study involving humans in accordance with the local legislation and institutional requirements. Written informed consent to participate in this study was not required from the participants or the participants’ legal guardians/next of kin in accordance with the national legislation and the institutional requirements.

## Author contributions

YL: Conceptualization, Data curation, Methodology, Writing – original draft, Writing – review & editing. MZ: Validation, Visualization, Writing – review & editing. WJ: Validation, Visualization, Writing – review & editing. LZ: Formal analysis, Funding acquisition, Methodology, Validation, Writing – review & editing. YW: Conceptualization, Funding acquisition, Methodology, Supervision, Writing – review & editing.

## Footnotes

^1^https://wwwn.cdc.gov/Nchs/Nhanes/2007-2008/UHM_E.htm

^2^http://www.cdc.gov/nchs/nhanes/index.htm

^3^https://www.R-project.org
