# Supplementary material for: Association of heavy metals exposure with lower blood pressure in the population aged 8–17 years: a cross-sectional study based on NHANES
Source: Front Public Health. 2024 Jul 5;12:1411123. doi: 10.3389/fpubh.2024.1411123 (PMC11259964; doi:10.3389/fpubh.2024.1411123)
Supplement: Supplementary file 1 [file Data_Sheet_1.pdf]

Supplementary Material

1 Supplementary Tables

Table S1. Comparison of urinary metal concentrations between hypertensive and non-hypertensive individuals among participants aged 6-18 years.

Metals(95%CI)[a]	Overall	No-hypertension	Hypertension	*P*-value	% above LOD[b]
	(n=2224)	(n=1991)	(n=233)		
Barium, μg/L	2.39(2.25,2.54)	2.40(2.24,2.55)	2.38(1.82,2.94)	0.960	99.55%
Cadmium, μg/L	0.08(0.08,0.09)	0.08(0.08,0.09)	0.08(0.07,0.10)	0.820	65.47%
Cobalt, μg/L	0.69(0.61,0.78)	0.71(0.62,0.80)	0.56(0.49,0.63)	**0.010**	99.82%
Cesium, μg/L	4.84(4.64,5.04)	4.89(4.69,5.09)	4.37(3.97,4.78)	**0.010**	100%
Molybdenum, μg/L	73.66(70.65,76.66)	73.48(70.19,76.76)	75.31(65.28,85.33)	0.740	100%
Lead, μg/L	0.40(0.37,0.42)	0.40(0.37,0.42)	0.38(0.32,0.43)	0.390	97.26%
Antimony, μg/L	0.08(0.08,0.09)	0.09(0.08,0.09)	0.08(0.07,0.09)	0.280	81.83%
Thallium, μg/L	0.20(0.19,0.21)	0.20(0.19,0.21)	0.18(0.16,0.20)	**0.030**	99.73%
Tungsten, μg/L	0.20(0.18,0.23)	0.21(0.18,0.23)	0.19(0.15,0.22)	0.390	95.64%
Uranium, μg/L	0.01(0.01,0.02)	0.01(0.01,0.01)	0.02(0.00,0.03)	0.450	88.04%
Mercury, μg/L	0.38(0.35,0.42)	0.39(0.35,0.42)	0.38(0.29,0.46)	0.840	76.35%
Arsenic, μg/L	11.62(9.50,13.74)	11.93(9.65,14.22)	8.74(6.62,10.86)	**0.030**	98.70%

[a] Weighted mean (95% confidence interval, 95%CI)
[b] The proportion of participants with urinary metal concentrations exceeding limits of detection (LOD).

Ba: barium; Cd: cadmium; Co: cobalt; Cs: cesium; Mo: molybdenum; Pb: lead; Sb: antimony; Tl: thallium; W: tungsten; U: uranium; Hg: mercury; As: arsenic.

Table S2. Associations of Ln-transformed urinary metal concentrations with hypertension based on the single metal model.

Characteristic	quartiles of urinary heavy metals				P for trend
	Q1	Q2	Q3	Q4	
Barium	ref	0.58(0.37,0.90)	0.81(0.47,1.39)	0.84(0.49,1.44)	0.833
Cadmium	ref	1.14(0.73,1.77)	0.66(0.38,1.14)	0.60(0.33,1.10)	0.318
Cobalt	ref	0.95(0.58,1.55)	0.65(0.36,1.18)	0.67(0.33,1.35)	0.324
Cesium	ref	0.82(0.47,1.42)	0.77(0.44,1.35)	0.57(0.28,1.15)	0.324
Molybdenum	ref	0.75(0.45,1.27)	0.90(0.54,1.48)	1.04(0.57,1.88)	0.833
Lead	ref	0.60(0.34,1.05)	0.61(0.37,1.02)	0.61(0.35,1.08)	0.324
Antimony	ref	0.59(0.35,1.01)	0.60(0.34,1.06)	0.48(0.23,0.96)	0.318
Thallium	ref	0.78(0.49,1.24)	0.76(0.41,1.42)	0.61(0.31,1.19)	0.331
Tungsten	ref	0.72(0.44,1.16)	0.74(0.49,1.12)	0.71(0.41,1.21)	0.356
Uranium	ref	0.68(0.44,1.03)	0.54(0.31,0.94)	0.64(0.33,1.22)	0.324
Mercury	ref	0.83(0.47,1.47)	0.73(0.37,1.44)	0.70(0.36,1.37)	0.382
Arsenic	ref	0.65(0.40,1.07)	1.02(0.63,1.63)	0.64(0.35,1.16)	0.382

The results are obtained using complex sampling weights and presented as weighted OR values (95% confidence interval, 95%CI).
Bold values indicate statistical significance P < 0.05.
P for trend were estimated by assigning an ordinal variable coded 1,2,3,4 for the quartiles of ln-transformed urinary metal levels as a categorical variable.
All P for trend were FDR-adjusted.
The models were adjusted for sex, age, race/ethnicity, family poverty income ratio (PIR), obesity, education, serum cotinine, serum creatinine, urinary creatinine, fish consumption, total energy, calcium intake, sodium intake, potassium intake and activity. Each metal predicts the risk of hypertension separately.

Table S3. Associations of Ln-transformed urinary metal concentrations with systolic and diastolic blood pressure based on the single metal model.

Characteristic	Q1	Q2	Q3	Q4	*P* for trend
			SBP, mmHg		
Barium	ref	-0.28(-1.75, 1.19)	1.04(-0.46, 2.54)	1.10(-0.25, 2.46)	0.117
Cadmium	ref	1.42(-0.11, 2.95)	-0.43(-1.65, 0.78)	-0.67(-2.43, 1.08)	0.300
Cobalt	ref	0.06(-1.41, 1.53)	0.34(-1.22, 1.91)	-1.48(-3.25, 0.28)	0.195
Cesium	ref	0.04(-1.60, 1.68)	-0.74(-2.53, 1.04)	-1.97(-4.43, 0.50)	0.195
Molybdenum	ref	0.62(-0.85, 2.09)	0.26(-1.23, 1.76)	-0.68(-2.57, 1.21)	0.511
Lead	ref	-1.68(-3.10,-0.27)	-2.19(-3.85,-0.52)	-3.29(-5.30,-1.28)	**0.036**
Antimony	ref	-0.27(-1.61, 1.08)	-0.73(-2.53, 1.07)	-1.73(-3.85, 0.40)	0.195
Thallium	ref	-0.77(-2.22, 0.69)	-0.63(-2.21, 0.95)	-1.64(-3.77, 0.49)	0.237
Tungsten	ref	1.06(-0.22, 2.34)	0.84(-0.52, 2.21)	0.37(-1.35, 2.08)	0.815
Uranium	ref	-0.09(-1.36, 1.18)	0.03(-1.73, 1.78)	-0.25(-2.30, 1.79)	0.849
Mercury	ref	0.69(-1.15, 2.54)	-0.49(-2.29, 1.31)	-1.58(-3.76, 0.60)	**0.036**
Arsenic	ref	-0.21(-1.48, 1.06)	0.01(-1.97, 1.99)	-2.09(-3.84,-0.34)	0.117
			DBP, mmHg		
Barium	ref	-2.23(-3.87,-0.59)	-1.87(-3.68,-0.06)	-1.53(-3.25, 0.18)	0.294
Cadmium	ref	0.23(-1.73, 2.20)	-1.93(-3.55,-0.32)	-0.39(-2.20, 1.43)	0.294
Cobalt	ref	‘-1.10(-3.25, 1.05)	-0.71(-2.52, 1.10)	-2.61(-4.62,-0.60)	0.084
Cesium	ref	-1.44(-3.18, 0.31)	-1.87(-3.90, 0.16)	-2.15(-4.69, 0.40)	0.196
Molybdenum	ref	-0.39(-2.08, 1.30)	-1.57(-3.45, 0.31)	-3.43(-5.37,-1.48)	**<0.001**
Lead	ref	-0.45(-2.01, 1.10)	-0.36(-1.99, 1.28)	-1.21(-3.27, 0.84)	0.382
Antimony	ref	-1.47(-3.28, 0.34)	-2.07(-4.22, 0.08)	-0.31(-2.30, 1.68)	0.698
Thallium	ref	-1.22(-3.08, 0.64)	-0.68(-2.32, 0.95)	-0.32(-2.63, 1.99)	0.937
Tungsten	ref	-0.9(-2.53, 0.72)	-2.88(-4.63,-1.13)	-1.60(-3.38, 0.19)	0.084
Uranium	ref	-0.91(-2.75, 0.93)	-1.23(-2.98, 0.52)	-1.87(-4.11, 0.36)	0.196
Mercury	ref	-0.99(-3.87, 1.88)	-1.52(-4.42, 1.39)	-2.32(-5.25, 0.60)	0.186
Arsenic	ref	-0.81(-2.53, 0.91)	-0.76(-2.94, 1.42)	-1.89(-3.99, 0.21)	0.295

SBP, systolic blood pressure; DBP, diastolic blood pressure.
The results are obtained using complex sampling weights and presented as weighted β values (95% confidence interval, 95%CI).
Bold values indicate statistical significance $P < 0.05$.
P for trend were estimated by assigning an ordinal variable coded 1,2,3,4 for the quartiles of ln-transformed urinary metal levels as a categorical variable.

All *P* for trend were FDR-adjusted.

The models were adjusted for sex, age, race/ethnicity, family poverty income ratio (PIR), obesity, education, serum cotinine, serum creatinine, urinary creatinine, fish consumption, total energy, calcium intake, sodium intake, potassium intake and activity. Each metal predicts blood pressure outcome separately.

Table S4. Distribution of speciated arsenic concentrations in the urine of children and adolescents in NHANES 2007–2016.

Metals(95%CI)[a]	Overall[b] (n=2207)	No-hypertension (n=1974)	hypertension (n=233)	*P*-value	% above LOD[c]	Miss (n)[d]
Arsenous acid	0.66(0.63,0.68)	0.66(0.64,0.69)	0.58(0.52,0.64)	**0.010**	37.43%	14
Arsenic acid	0.64(0.63,0.65)	0.64(0.63,0.65)	0.64(0.62,0.65)	0.370	2.31%	16
Arsenobetaine	5.10(3.11,7.08)	5.32(3.16,7.47)	3.06(1.74,4.38)	**0.040**	33.53%	15
Arsenocholine	0.26(0.24,0.28)	0.26(0.25,0.28)	0.24(0.21,0.26)	0.110	6.43%	14
Dimethylarsonic acid	4.54(4.20,4.89)	4.59(4.23,4.96)	4.08(3.27,4.88)	0.230	79.25%	14
Monomethylacrsonic acid	0.76(0.72,0.80)	0.77(0.72,0.81)	0.69(0.61,0.76)	0.050	45.27%	14

[a] Weighted mean (95% confidence interval, 95%CI).
[b] Among the 2224 children and adolescents enrolled in our study, 17 participants with missing speciated arsenic concentrations were excluded.
[c] The proportion of participants with urinary metal concentrations exceeding limits of detection (LOD).
[d] The count of participants with speciated arsenic concentrations missing
Bold values indicate statistical significance $P < 0.05$.

Table S5. Association of speciated arsenic concentrations with blood pressure

	WQS mixture result[a]	*P*-value	Component (weights)[b]
Hypertension	-0.09(-0.56, 0.38)	0.718	NA
SBP	-0.88(-1.62， -0.13)	**0.020**	As(III) (40.00%), AsC (34.04%)
DBP	-0.86(-1.84, 0.12)	0.087	NA

[a] After adjusting for potential confounding factors, including sex, age, race/ethnicity, family poverty income ratio (PIR), obesity, education, serum cotinine, serum creatinine, urinary creatinine, fish consumption, total energy, calcium intake, sodium intake, potassium intake and activity, the weighted quantile sum regression (WQS) model was employed to investigate the influence of Ln-transformed speciated arsenic concentrations on blood pressure. The estimated parameters (OR_{index} or β_{index}), their corresponding 95% confidence intervals (CI), and *P*-values were reported for each model.

[b] The component (weights) represent the metals in the model that exhibit significant effects and indicate their corresponding percentages.

Bold values indicate statistical significance $P < 0.05$.

SBP, systolic blood pressure; DBP, diastolic blood pressure; NA: not applicable.

Table S6. Associations of urinary metals with blood pressure among study participants with urinary creatinine values between 30 – 300 mg/dL (n=2,022).

	WQS mixture result[a]	*P*-value	Component (weights)[b]
Hypertension	0.21(-0.26, 0.68)	0.370	NA
SBP	-0.88(-1.68, -0.08)	**0.010**	As (32.4%), Sb (21.6%), Cd (18.7%), Cs(8.87%)
DBP	-1.40(-2.75, -0.48)	**0.044**	Cs (31.78%), Cd (17.53%), W (14.07%), As(13.43%)

[a]After adjusting for potential confounding variables, including sex, age, race/ethnicity, family poverty income ratio (PIR), obesity, education, serum cotinine, serum creatinine, urinary creatinine, fish consumption, total energy, calcium intake, sodium intake, potassium intake and activity, we employed the weighted quantile sum regression (WQS) model to examine the impact of Ln-transformed urinary metal concentrations on blood pressure in participants with urinary creatinine values ranging from 30 to 300 mg/dL (n=2,022). We reported the estimated parameters (OR_{index} or β_{index}), their corresponding 95% confidence intervals (CI), and *P*-values for each model.

[b] The component (weights) represent the metals in the model that exhibit significant effects and indicate their corresponding percentages.

Bold values indicate statistical significance $P < 0.05$.

SBP, systolic blood pressure; DBP, diastolic blood pressure; NA: not applicable.

2 Supplementary Figures

Fig S1. Flow chart of the participant selection.

Fig S2. Pearson correlation matrix for twelve urinary metals.

Fig S3. Association of metal mixture with blood pressure estimated by Bayesian Kernel Machine Regression (BKMR) among 2224 children and adolescents aged 8–17 years from NHANES 2007–2016.

A, B, C: The convergence plots for single parameters were generated by fitting metal mixtures to investigate their effects on hypertension, systolic blood pressure, and diastolic blood pressure using the BKMR model. The results provide confirmation of the convergence of the BKMR model (what we expect here is some kind of random behaving around a straight line).

D, F, H: Univariate exposure-response function and 95% confidence interval for each toxicant with the other toxicants fixed at the median. D, F, and H represent hypertension, systolic blood pressure, and diastolic blood pressure, respectively.

E, G, I: Single toxicant effect on blood pressure comparing the upper quartile to the lower quartile level of a particular toxicant while fixing the other toxicants at the 25th,50th, and 75th percentile. E, G, and I represent hypertension, systolic blood pressure, and diastolic blood pressure, respectively.

The models were adjusted for several potential confounders, including sex, age, race/ethnicity, family poverty income ratio (PIR), obesity, education, serum cotinine, serum creatinine, urinary creatinine, fish consumption, total energy, calcium intake, sodium intake, potassium intake and activity.

Fig S4. The differential association between metal mixtures and blood pressure based on Bayesian Kernel Machine Regression (BKMR) among fish consumers and non-fish consumers.

A, C, and E represent hypertension, systolic blood pressure, and diastolic blood pressure among non-fish consuming children and adolescents, while B, D, and F represent the same parameters in fish consuming children and adolescents.

Fig S5. The weights of each metal in WQS model regression index for blood pressure among study participants with urinary creatinine values between 30 – 300 mg/dL (n=2,022).

A, B, and C display the weights of each metal in WQS model regression index for hypertension, systolic blood pressure, and diastolic blood pressure.
