# Supplementary figures and images for: Association of heavy metals exposure with lower blood pressure in the population aged 8–17 years: a cross-sectional study based on NHANES
Source: Front Public Health. 2024 Jul 5;12:1411123. doi: 10.3389/fpubh.2024.1411123 (PMC11259964; doi:10.3389/fpubh.2024.1411123)

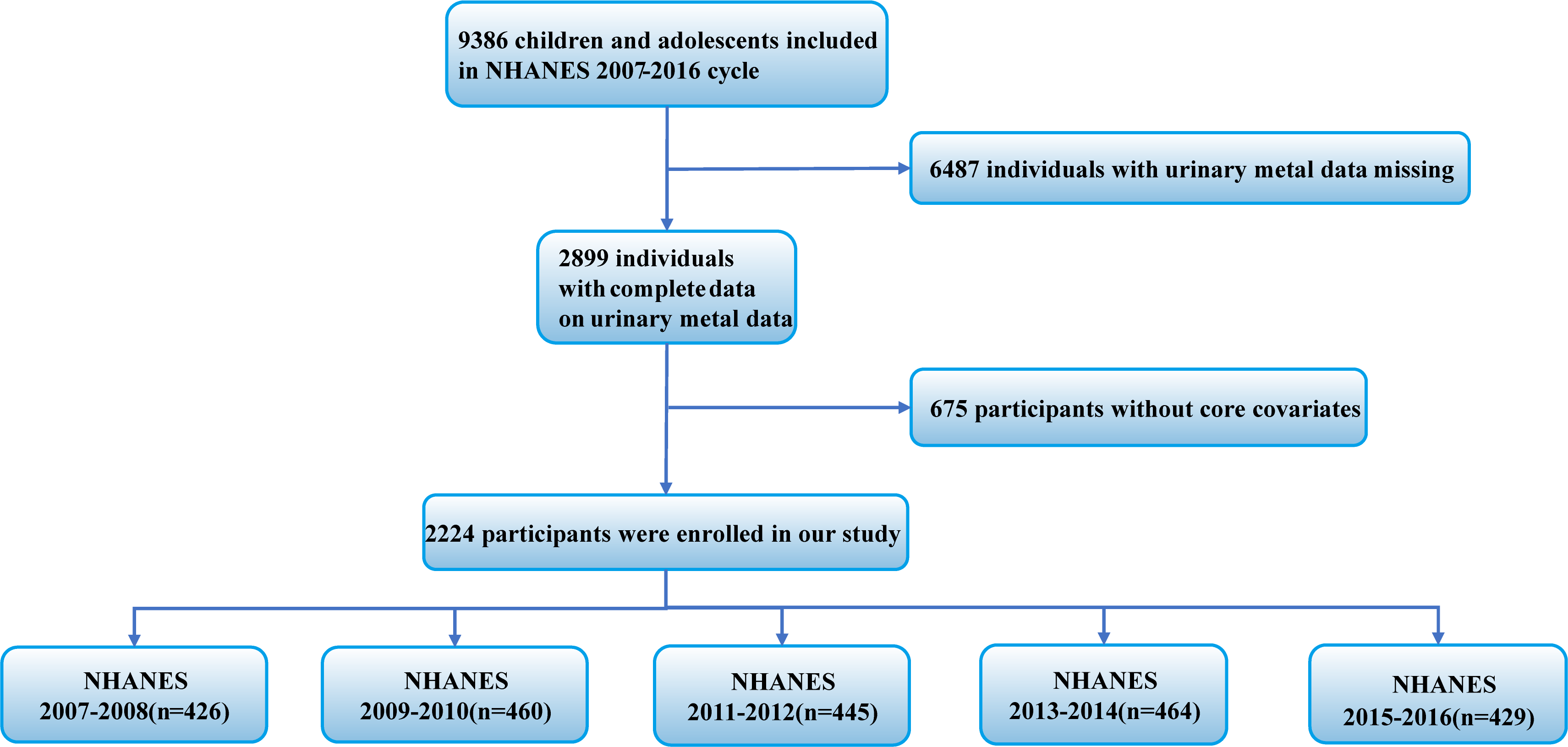

Supplement: Supplementary file 2 [file Image_1.TIF]

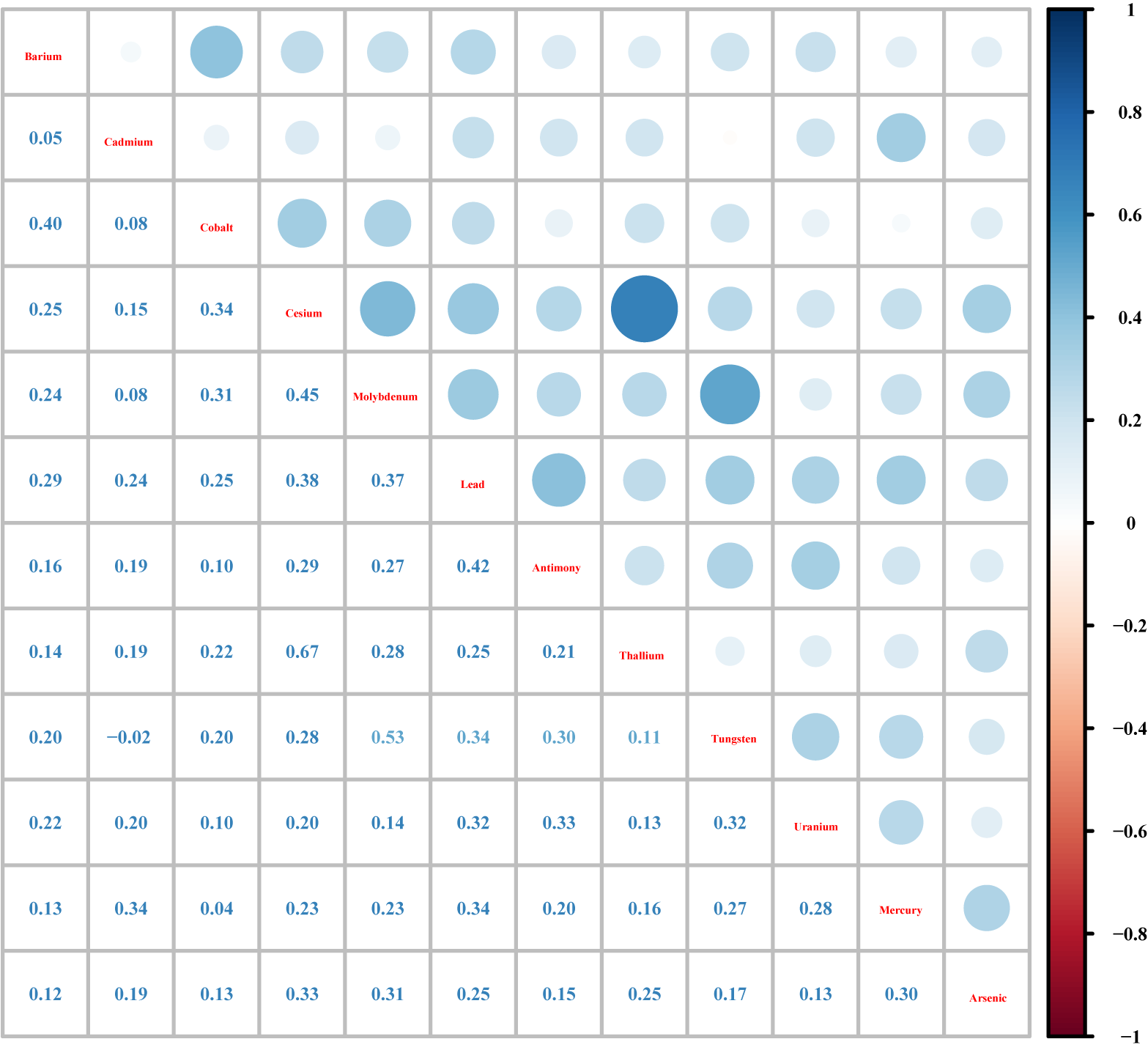

Supplement: Supplementary file 3 [file Image_2.TIF]

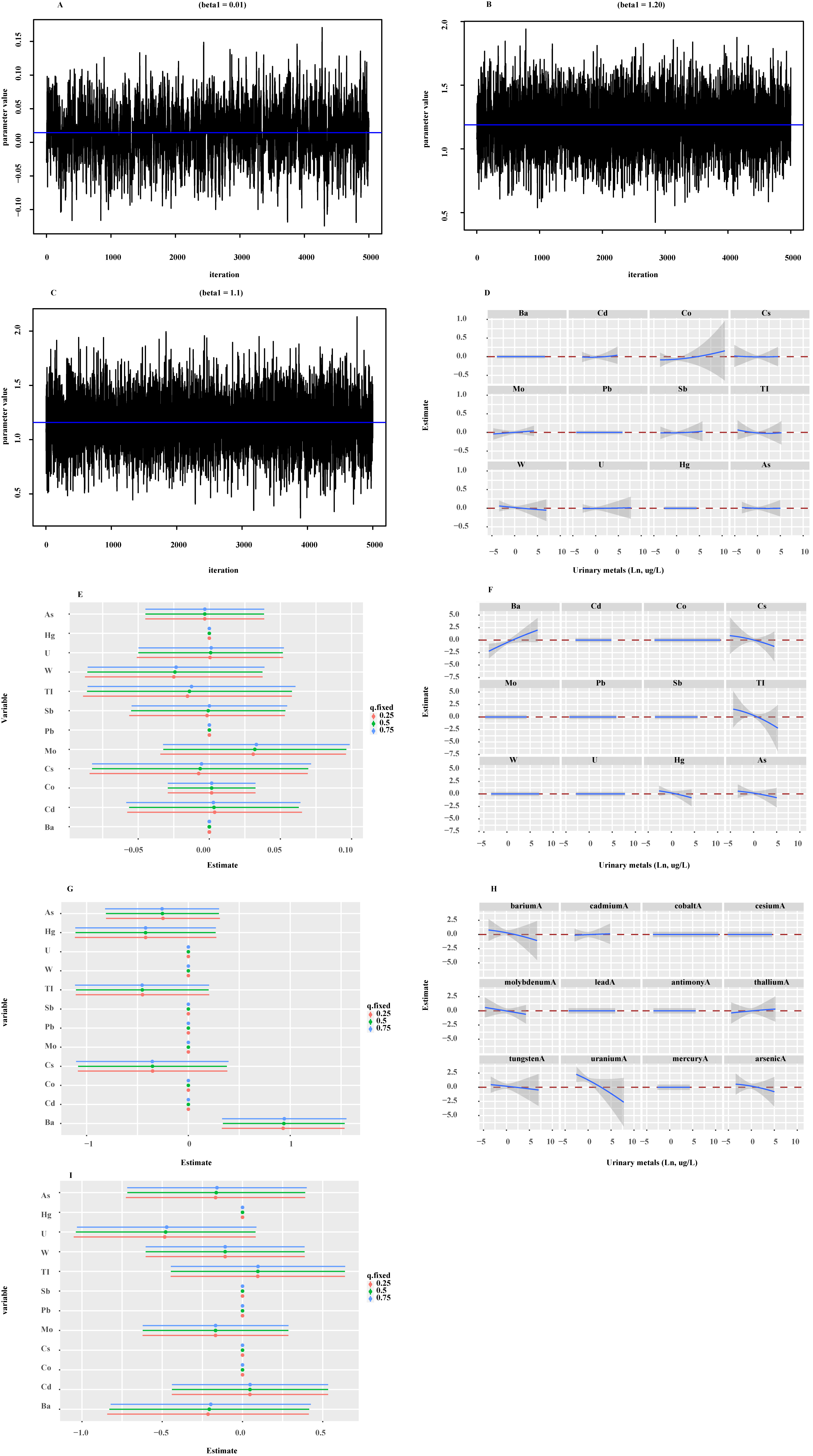

Supplement: Supplementary file 4 [file Image_3.TIF]

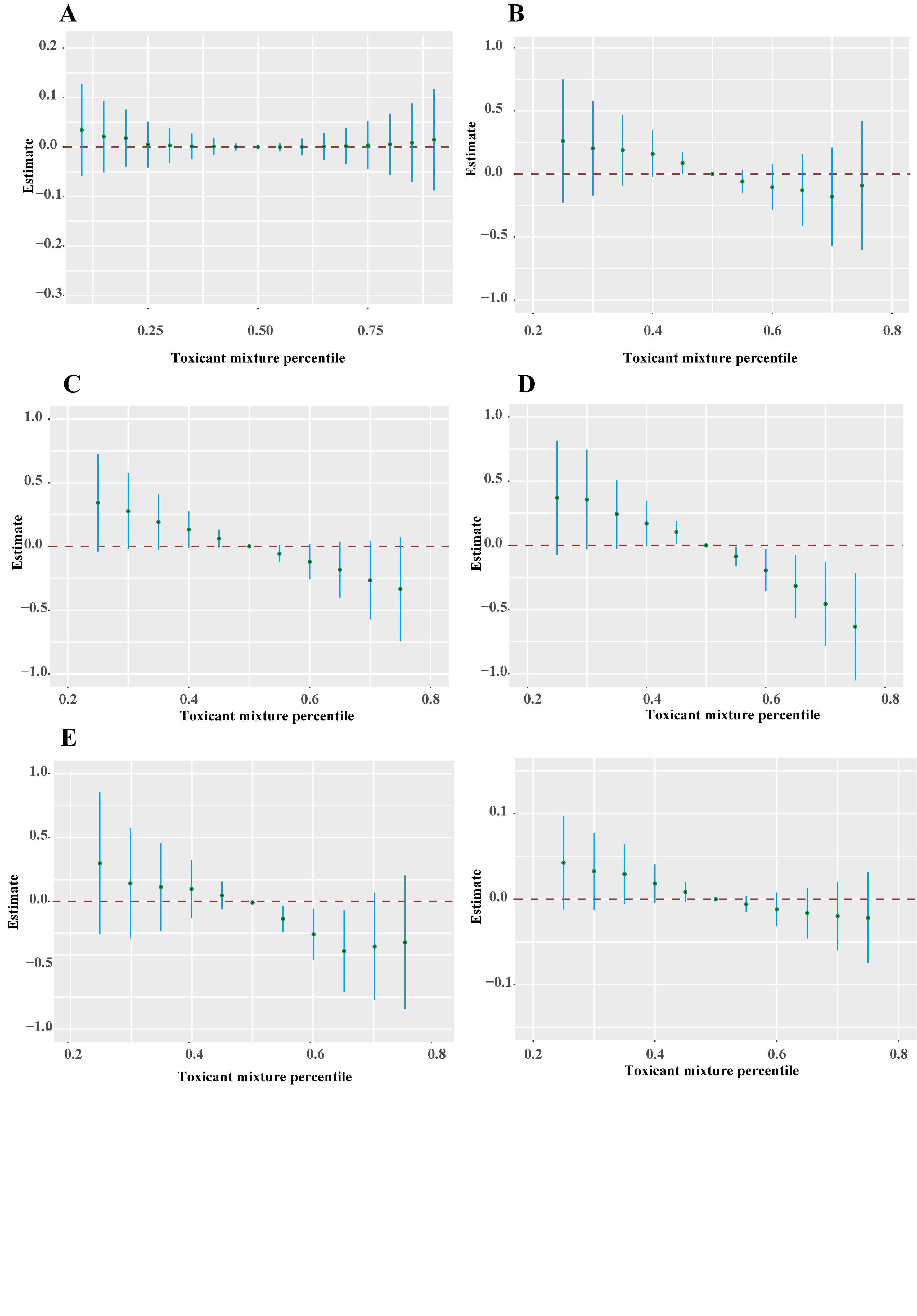

Supplement: Supplementary file 5 [file Image_4.TIF]

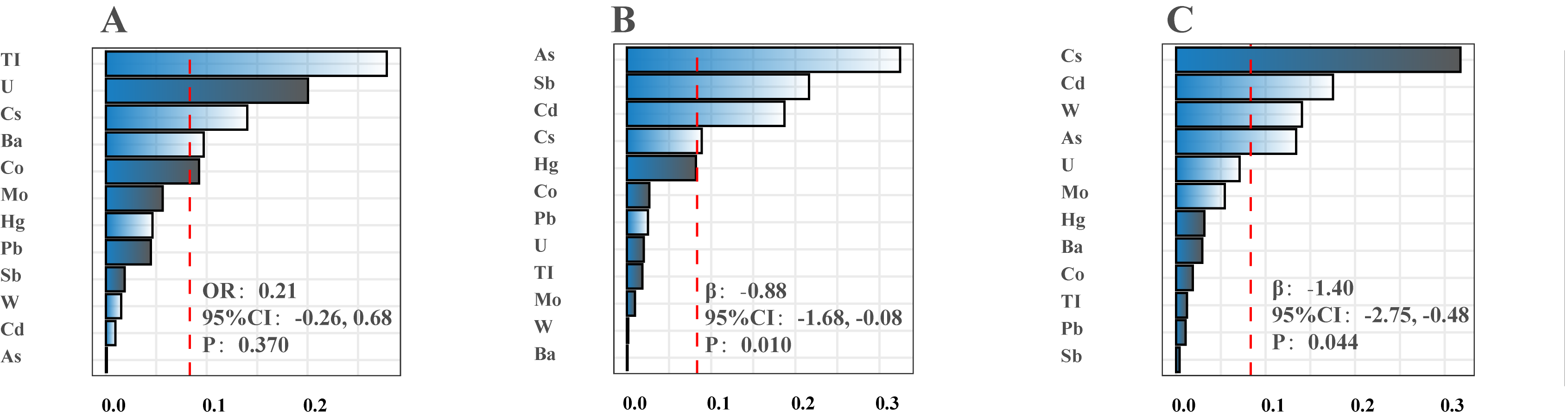

Supplement: Supplementary file 6 [file Image_5.TIF]
